# Molecular Characterization and Expression Profiling of Three *Transformer-2* Splice Isoforms in the Redclaw Crayfish, *Cherax quadricarinatus*

**DOI:** 10.3389/fphys.2020.00631

**Published:** 2020-07-09

**Authors:** Lina Cai, Jianbo Zheng, Yongyi Jia, Zhimin Gu, Shili Liu, Meili Chi, Shun Cheng

**Affiliations:** ^1^Key Laboratory of Freshwater Aquatic Genetic Resources, Ministry of Agriculture, Shanghai Engineering Research Center of Aquaculture, National Demonstration Center for Experimental Fisheries Science Education, College of Fisheries and Life Science, Shanghai Ocean University, Shanghai, China; ^2^Key Laboratory of Genetics and Breeding, Zhejiang Institute of Freshwater Fisheries, Huzhou, China

**Keywords:** *Cherax quadricarinatus*, *Tra-2*, gene expression, sex determination, RNAi

## Abstract

Sex determination/sex differentiation is determined by genetics, environmental factors, or the interactions of the two. The *Transformer-2* (*Tra-2*) gene plays an important role in the sex determination cascade signal pathway in insects. In this study, the *Tra-2* gene was isolated and characterized from the cDNA library of gonad tissues in the redclaw crayfish, *Cherax quadricarinatus.* Three splice variants were identified, designated as *CqTra-2A, CqTra-2B*, and *CqTra-2C*, and sequence analysis showed that they had a highly conserved RRM domain. Phylogenetic analysis was performed by the NJ method, and the results revealed that the Tra-2 protein of the redclaw crayfish was very closely related to those of *Macrobrachium rosenbergii*, *Fenneropenaeus chinensis*, and *Macrobrachium nipponense.* Real-time PCR analysis showed that the three isoforms were predominantly expressed in the ovary and gradually increased with embryonic development. Additionally, the expression pattern of *CqTra-2* at different developmental stages was analyzed by qPCR and revealed that the phase of having a body length of 3 cm may be the key period for the sex differentiation of *C. quadricarinatus.* RNAi-targeting gene silencing further confirmed the function of *CqTra-2* in sexual differentiation in redclaw crayfish. Our experimental data will contribute to understanding the mechanism of sex determination in crustaceans.

## Introduction

Decapod aquaculture is one of the most important economic industries in China and brings large benefits to farmers. Interestingly, in crustaceans, many biological or economic traits exist that are significantly different between males and females, such as growth rate and body size (Sagi et al., 1986; Browdy, 1998; Yu et al., 2017). Therefore, having an all-male or all-female culture of aquatic animals plays an important role in increasing yield and improving quality based on sexual control technology. For instance, all-male *Macrobrachium rosenbergii* can be generated successfully through manipulation of the insulin-like androgenic gland (Sagi et al., 1990; Lezer et al., 2015), which would enable the total worldwide farmed volume to reach more than 200,000 tons. However, studies on the regulatory mechanism of sex determination in crustaceans are still limited at present, which impedes the development of sex-controlled breeding techniques.

The molecular mechanism of sex determination and differentiation in crustaceans is highly complex and currently remains poorly understood, which is likely due to the rapid evolution of genes involved in this process (Zhang and Qiu, 2010). To date, many sex-related genes in crustaceans have been reported. For example, a body of evidence demonstrates that the insulin-like androgenic gland hormone gene (*IAG*) is the key factor that drives male sexual differentiation in crustacean species (Sagi et al., 1990; Shi et al., 2019). In addition, the expression of *Dmrt* genes is affected by silencing the *IAG* gene, suggesting a possible role in the sex-differentiation processes (Amterat Abu Abayed et al., 2019). Several female sexual differentiation genes, *foxl2*, *fem1*, and *cfsh*, have also been investigated in different crustacean species (Ma et al., 2016; Jin et al., 2018; Jiang et al., 2020).

The redclaw crayfish, *Cherax quadricarinatus*, is native to the tropical regions of northern Australia and southern New Guinea (Ventura et al., 2018). After being introduced into China in the 1990s, it was favored by domestic breeding enterprises and consumers. Like other crustaceans, the redclaw crayfish exhibited sexual dimorphism in growth traits, with males growing faster and bigger than females at harvest (Zheng et al., 2019). In addition, the female redclaw crayfish holds fewer eggs than other shrimps, which is one of the major factors affecting the development of the industry. The above status indicates that developing monosex breeding for producing all-female or male offspring can meet the requirements of actual production. Therefore, a better understanding of the sex determination and differentiation mechanisms of *C. quadricarinatus* to achieve sex-controlled breeding is urgently required.

Genetic studies have shown that crustaceans are evolutionarily closely related to insects in evolution (Budd and Telford, 2009), and the “*Sxl-tra/tra2-dsx*” cascade signaling pathway has been shown to play an important role in sex development in *Drosophila melanogaster* (Inoue et al., 1990; Penalva and Sanchez, 2003). To date, *Tra-2* gene has been cloned and analyzed in several crustacean species, including *Penaeus monodon* (Leelatanawit et al., 2009), *Fenneropenaeus chinensis* (Li et al., 2012), *Daphnia pulex* (Chen et al., 2014), *Eriocheir sinensis* (Luo et al., 2017), and *Macrobrachium nipponense* (Wang et al., 2019). Although the nucleotide sequences of *Tra2* genes are similar to that of *D. melanogaster*, their alternative splicing patterns and expression specificity are quite different. These studies suggest that crustaceans regulate the sex differentiation mechanism differently than insects, which is dependent on alternative splicing of pre-mRNA in a series of genes involved in the sex determination pathway.

In the present study, three mature *tra2* mRNA splice variants were identified from *C. quadricarinatus*, which were designated as *CqTra-2A*, *CqTra-2B*, and *CqTra-2C*. The nucleotide and amino acid characterization of the three *tra2* homologs are reported. Their expression distribution in various tissues and at various embryonic stages and different juvenile developmental periods were analyzed. Moreover, the effect of *CqTra2* gene silencing via RNA interference (RNAi) on the expression of *Cqdsx* was investigated. These results will be helpful for understanding the progress of the sex regulation mechanisms of crustaceans.

## Materials and Methods

### Animals and Samples Collection

The redclaw crayfish used in this experiment were taken from the Balilidian breeding base of the Zhejiang Institute of Freshwater Fisheries (Huzhou, Zhejiang Province). The average body weight and body length were 60 ± 0.5 g and 14.5 ± 0.2 cm, respectively. Tissues, including the heart (Hea), hepatopancreas (Hep), ovary (Ov), testis (Te), muscle (Mu), intestine (In), and gill (Gi), were dissected from three males and three females. Embryos at different developmental stages, including fertilized eggs (I), cleavage and blastula (II), gastrula (III), nauplius (IV), eye pigments forming (V), and prehatching (VI), were collected from gravid crayfish. Description of the embryonic development of C. *quadricarinatus* was based on a previously published method (Meng et al., 2001). Juveniles that cultured in the pool of a greenhouse were collected at different body length stages. All the samples were immediately frozen in liquid nitrogen and transferred to −80°C for nucleic acid extraction.

### RNA Extraction and cDNA Synthesis

Total RNA were extracted from various tissues and embryonic samples using Trizol Reagent (KangWei, Beijing) according to the manufacturer’s protocol. The RNA quality was detected by electrophoresis on 1% agarose gel, and the concentration was measured by NanoDrop 2000c (Thermo Scientific, United States). The isolated RNA was treated with RNase-free *DNase I* (Promega, United States). Approximately 1 μg RNA was used to synthesize cDNA using the HiFiScript cDNA First-strand Synthesis Kit (KangWei, Beijing).

### Full-Length cDNA Identification and Partial Genomic DNA Amplification

Genes encoding putative Tra2 proteins were screened from the transcriptomes of *C. quadricarinatus* gonadal tissues that were constructed in our lab (not published) by using *F. chinensis* Tra2 sequences (accessions: JQ239126, JQ239127, and JQ239128) as a query. Three sequences were found to be similar to members of the *tra2* homolog in *F. chinensis*. Additionally, three pairs of gene-specific primers were designed to obtain the full-length transcripts of three *Cqtra2* homologs. PCRs using the cDNA template from the ovary were performed under the following conditions: one cycle of 95°C for 5 min; 32 cycles including denaturation at 95°C for 30 s, annealing at 56°C for 30 s, and extension at 72°C for 90 s; one cycle of 72°C for 7 min. The PCR products were separated using 1.0% agarose gels, ligated to the pMD18-T vector (TAKARA, Japan), and transformed into *Escherichia coli* competent cells (KangWei, Beijing) for sequencing.

Furthermore, the partial genomic sequence of *CqTra2* was cloned using a DNA template that was extracted from ovarian tissue via the genome walking method according to the protocol of the GenomeWalker Universal Kit (Clontech, Japan). Specific reverse primers (SRP1 and SRP2) were designed from previously obtained cDNA sequences, and the method for obtaining, purifying, and sequencing PCR products was as previously described. All primers used in this section are shown in Table 1.

**TABLE 1:** Primers used for gene expression analysis of *CqTra-2* in *C. quadricarinatus.*

Primer name	Sequences (5′-3′)	Purpose
Full-T*CqTra-2A-*F	CGCCTGTGTTCCTGAGTACTTAGGT	Full-length validation
Full-T*CqTra-2A-*R	TACTCTTCTCAATCACTACCATCTC	Full-length validation
Full-T*CqTra-2B-*F	CTGTTGCCACACTAGGTATGAGCGA	Full-length validation
Full-T*CqTra-2B-*R	ACCTCTATTCCACTTTGACAGCGGG	Full-length validation
Full-T*CqTra-2C-*F	AGCGAGTCTCCAGGTGGTGGCAGTA	Full-length validation
Full-T*CqTra-2C-*R	TGGTTTCATCTTACACTCAGTTTTC	Full-length validation
SRP1	AAATCTCTTCTTCAAACCAACACCGAA	Genome walking
SRP2	CCTTCTGTAAACACGATAATGACCGAG	Genome walking
*CqTra-2A-*F	TAGACCCACATATTCTGGTTAT	qRT-PCR
*CqTra-2A-*R	CACCATTACTTCATTCTTCA	qRT-PCR
*CqTra-2B-*F	CTGGTTATGGAAGACGTGGT	qRT-PCR
*CqTra-2B-*R	TCAATACCGGCTGTTGCGAC	qRT-PCR
*CqTra-2C-*F	GAGATTGACGGCCGACGCA	qRT-PCR
*CqTra-2C-*R	CAGTTTTCAAGAGCGTAACTCCA	qRT-PCR
*Cq-18S*-F	CTGAGAAACGGCTACCACATC	qRT-PCR
*Cq-18S*-R	GCCGGGAGTGGGTAATTT	qRT-PCR
*Cq*-*dsx*-F	TGGCACTCTGGTCCAGGAAGG	qRT-PCR
*Cq*-*dsx*-R	CCGTCGTCGTCATCAGCAGTAG	qRT-PCR
*CqTra-2-*F	ACGGAACATCTCCCATTC	qRT-PCR
*CqTra-2-*R	TACTTGTCGTCGCGGCA	qRT-PCR

### Bioinformatics Analyses

The open reading frame and amino acid sequences of *CqTra-2* were predicted using online software^1^. The secondary structure was analyzed by GOR4^2^, and multiple alignments among the different Tra-2 proteins were carried out by BLAST^3^. The phylogenetic tree was constructed using the Neighbor-Joining method and MEGA 5.0 software. The amino acid sequences of Tra-2 from other species (Table 2) were obtained from the NCBI website.

**TABLE 2:** Tra2 proteins used for phylogenic analysis.

Species	GenBank No.	Species	GenBank No.
*Cherax quadricarinatus*	MN393520	*Ceratitis capitata*	NP_001266337
*Cherax quadricarinatus*	MN393521	*Daphnia magna*	JAM95870.1
*Cherax quadricarinatus*	MN393522	*Lucilia cuprina*	XP_023305738
*Fenneropenaeus chinensis*	JQ239126	*Macrobrachium rosenbergii*	QBY91826
*Fenneropenaeus chinensis*	JQ239127	*Macrobrachium rosenbergii*	QBY91827
*Fenneropenaeus chinensis*	JQ239128	*Eriocheir sinensis*	KU291992
*Bombyx mori*	NP_001119709	*Eriocheir sinensis*	KU291993
*Drosophila melanogaster*	P19018	*Eriocheir sinensis*	KU291994
*Apis mellifera*	NP_001252514	*Eriocheir sinensis*	KU291995
*Aedes aegypti*	AAEL006416	*Penaeus monodon*	ACD13597
*Macrobrachium nipponense*	AGI50962		

### Expression Profile Detected by Quantitative Real-Time PCR (qRT-PCR)

qRT-PCR assays were employed to measure gene expression levels using cDNA that was prepared from different tissues, embryos, and individuals with different body lengths. The specific primers were designed for three mature *tra-2* isoforms (Table 1). qRT-PCR was performed on a LightCycler^®^480 system (Roche, Switzerland) in a 10-μL reaction mix containing 1 μL of cDNA template, 0.5 μL of each primer (10 μM), 3 μL of water, and 5 μL of SYBR Premix Ex Taq^TM^ (TaKaRa, China). *18S-rRNA* was used as the reference gene, and each test was performed in triplicate. The melting curves were analyzed after amplification to identify the specific product in all PCRs. The threshold cycle (Ct) values via the 2^–ΔΔCt^ method were calculated using the qRT software provided for the LightCycler^®^ 480 System (Tan et al., 2017). A histogram for fold comparison of different samples was generated by inputting the 2^–ΔΔCt^ values of different samples into the GraphPad Prism 5 program software (Roche, Switzerland). Statistical analyses were performed by SPSS 19.0, and significant differences were determined using a Pearson test.

### Knock-Down of *Cqtra2* by dsRNA-Mediated RNA Interference

Fragments of *CqTra-2* (350 bp) and *EGFP* (359 bp) containing T7 promoter were synthesized by the GenScript company and subcloned into the vector pUC57. The recombinant plasmids were digested at *Hin*dIII or *Eco*RI, and the purified product was used as the template for dsRNA synthesis with a MEGA script RNAi kit (Thermo Fisher Scientific, United States). For *in vivo* gene knockdown, 5 μg/g dsRNA was injected by microsyringe (0–50 μL, Ningbo Zhenhai Sanai instrument factory) into a single undifferentiated crayfish (juvenile crayfish in which the secondary sex characteristics could not be distinguished) (*n* = 10) with an average weight of 0.2 g and average body length of 2 cm. After injection, all crayfish were returned to the culture tanks for 2 weeks until the cephalothoraxes were dissected. Total RNA was extracted, and the first-strand cDNA synthesis of each sample was carried out as described above. To determine the RNAi effect, the expression level of *Cqdsx* (Accession No. MK342618) was investigated by qRT-PCR quantification, using the qPCR method described above. Three replicates were used for analysis. The results were expressed as the mean ± SEM, and a Student’s *t*-test was used to analyze the difference between groups.

### Statistical Analyses

Statistical analyses were performed using SPSS software version 13.0. Data were expressed as mean ± SD (*n* = 3), and statistical significance was determined by one-way ANOVA. Significance was set at *P* < 0.05.

## Results

### Molecular Identification and Sequence Analysis

**FIGURE 1:**
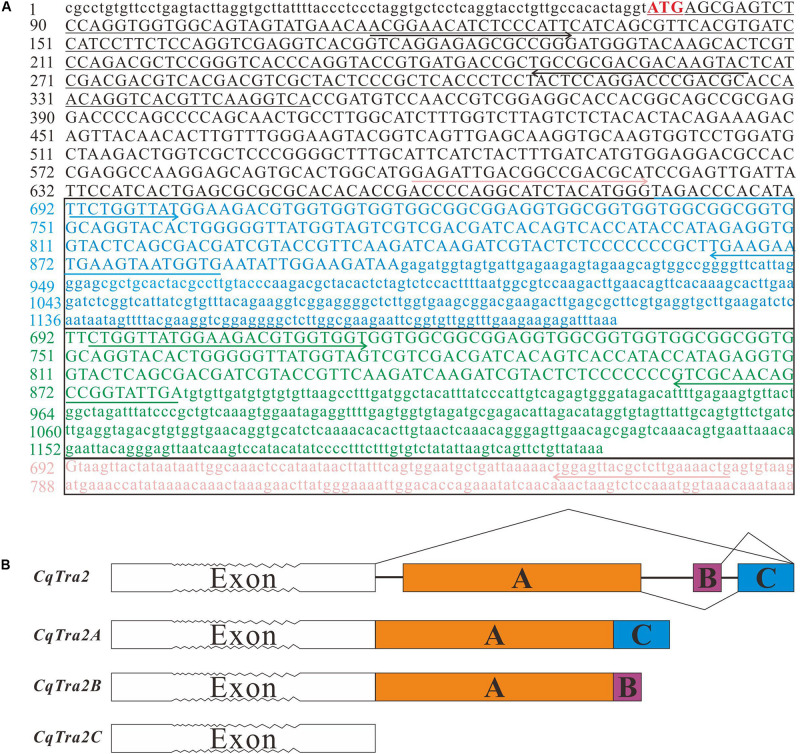
Nucleotide sequences and schematic diagram of *Tra2* homologs in *C. quadricarinatus*. **(A)** Nucleotide sequences of Tra2 homologs (*CqTra-2A*, *CqTra-2B*, and *CqTra-2C*). The start codon (ATG) is marked with a red background. The nucleotides are numbered on the left. The lower-case letters indicate 5′ and 3′ untranslated regions, and upper-case letters indicate the coding region. The different colors represent the specific region of the three spliced isoforms, in which blue represents *CqTra-2A*, green represents *CqTra-2B*, and orange represents *CqTra-2C*. The different colored solid line arrows show the primers used in RT-PCR for the three different isoforms. Underlining represents the sequence used to synthesize the dsRNA for RNAi. **(B)** Schematic representation of the genomic sequence of *CqTra2* and pre-mRNA splicing. White boxes with serrated teeth indicate mutual exons but not exact numbers of exons. Colored boxes show the alternatively spliced exons. The solid lines show introns, the crease lines show the manner of alternative splicing of *Cqtra2* pre-mRNA.

By searching homologous sequences submitted to the NCBI GenBank, the full-length cDNA sequence of *Tra-2* was obtained from the transcriptome database constructed in our lab and validated by Sanger sequencing. As a result, three different *Tra-2* isoforms were identified from a cDNA library of gonad tissues, including *CqTra-2A*, *CqTra-2B*, and *CqTra-2C*. The full-length cDNA of *CqTra-2A* was 1207 bp, containing an 822 bp open reading frame (ORF) that encoded 273 amino acids. *CqTra-2B* was 1225 bp in length, with an 804 bp ORF encoding 267 amino acids. The full-length *CqTra-2C* cDNA sequence consisted of 880 bp nucleotides with 615 ORF and 204 amino acids (Figure 1A). We have submitted the full-length cDNA sequences of *CqTra-2* to GenBank (Accession no. MN393520, MN393521, and MN393522).

A large number of repeat sequences existed in the intron region during genomic DNA amplification using Genome Walking in *C. quadricarinatus*, presenting a great obstacle to obtaining the full-length *Tra2* sequence. Sequence alignment analysis revealed that three *Cqtra2* homologs had a consistent 5′-UTR, and their difference chiefly appeared in the terminal region of the cDNA sequence. Therefore, the gene structures with a partial exon-intron organization pattern were analyzed from the different sites to the gene terminal were analyzed (data from DNA sequence not shown). Sequence analysis revealed that the three *CqTra2* cDNA isoforms were in the same genomic locus, with differences in the splice pattern of the last three exons. As shown in Figure 1B, *CqTra-2A* contained exon A and exon C, and *CqTra-2B* contained exon A and exon B, while the three exons were absent from *CqTra-2C*.

### Multiple Alignment and Phylogenetic Analysis

**FIGURE 2:**
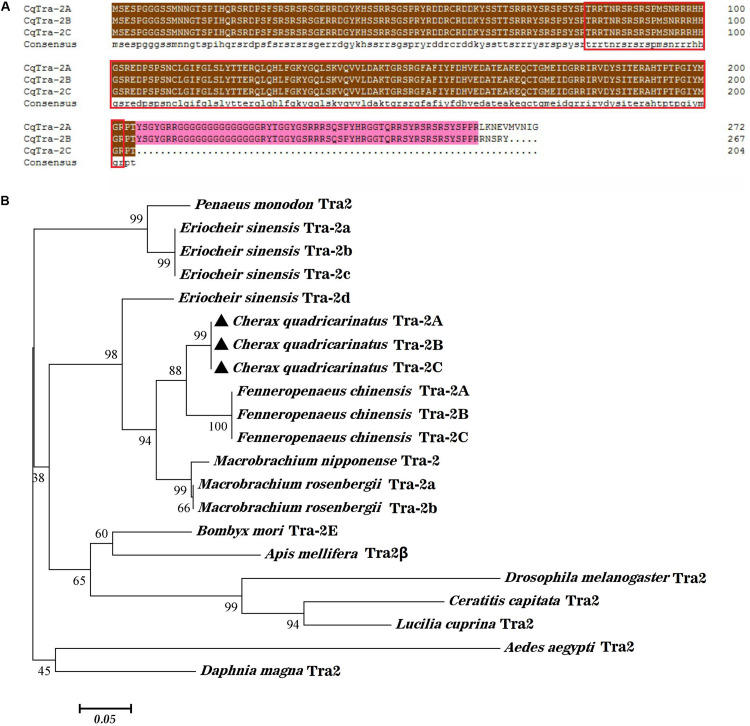
Amino acid alignment and phylogenetic tree analysis of Tra-2. **(A)** Sequence alignment of three isoforms of Tra-2 in *C. quadricarinatus*. Red rectangle indicates the RRM domain. **(B)** Phylogenetic tree construction of Tra-2 protein (Neighbor-Joining method). The black triangle shows the *C. quadricarinatus* species, and numbers in the branches represent the bootstrap values (%) from 1000 replicates. Species names and Tra2 protein types are listed on the right of the tree.

The multiple alignment of amino acid sequences by BLAST showed that the identity of all homologs of CqTra2 was very high, and domain prediction using the online software GOR4 revealed that they all possessed a highly conserved RNA recognition motif (RRM) that was shared with other Tra2 proteins (Figure 2A). Based on the clustalW algorithm alignment of 21 Tra-2 members (Table 2), the phylogenetic tree was constructed by the neighbor-joining method with 1000 bootstrap replicates using MEGA 5 software. The phylogenetic tree showed that the Tra2 protein was highly conserved among shrimp and crab species, including *C. quadricarinatus*, *M. rosenbergii*, *F. chinensis*, *E. sinensis*, and *M. nipponense*. Curiously, *D. pulex*, part of the scientific class Crustacea, was classified into a single clade with Tra-2 sequences from *Aedes aegypti* that belonged to insects (Figure 2B).

### Tissue-Specific Distribution of Three *CqTra2* Transcripts in Adult Crayfish

**FIGURE 3:**
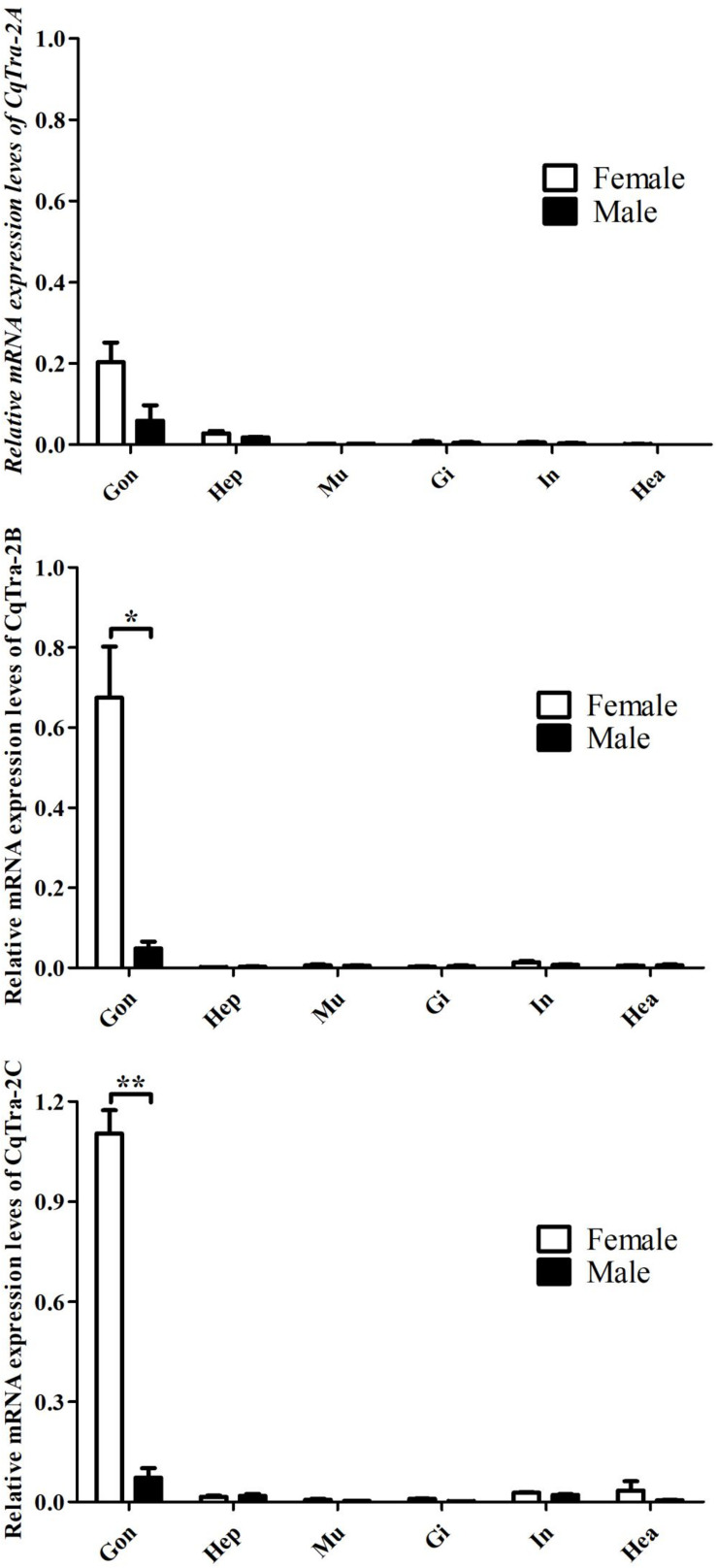
Distribution of three CqTra-2 isoforms in various tissues from C. quadricarinatus. The 18S-rRNA gene is used as control. Gon, gonads (Ovary and Testis, respectively); Hep, hepatopancreas; Mu, muscle; Gi, Gill; In, intestine; Hea, heart. Results are expressed as mean ± SEM, and significance of comparison is defined as *P* < 0.05 (*) or *P* < 0.01 (**) by Student’s *t*-tests.

A quantitative real-time PCR assay was used to detect the expression level of three different splice variants of *CqTra-2* in adult tissues. Tissue distribution analysis revealed that the three splice isoforms were all highly expressed in the ovary and weakly expressed in the testis, while low expression was observed in other tissues, including the hepatopancreas, muscle, gill, intestine, and heart (Figure 3). In the gonads, expression profiling of *CqTra-2B* and *CqTra-2C* showed sex differences with a higher expression level in the ovary than in the testis (*P* < 0.05), but *CqTra-2A* mRNA abundance was not significantly different between female and male gonads (*P* > 0.05). Additionally, *CqTra-2* expression did not significantly differ between adult male and female tissues of *C. quadricarinatus.*

### Expression Pattern in Different Developmental Stages of Embryos and Juveniles

**FIGURE 4:**
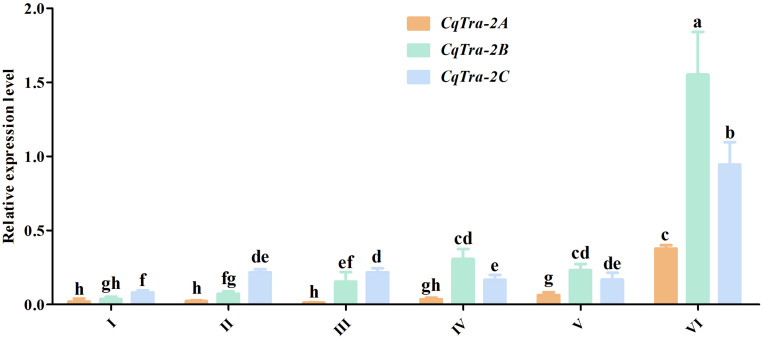
Expression pattern of *CqTra-2* genes in different embryonic development stages of *C. quadricarinatus*. The *18S-rRNA* gene is used as control. (I) Fertilized eggs, (II) cleavage and blastula, (III) gastrula, (IV) nauplius, (V) eye pigments forming, and VI) prehatching. Data are shown as means ± SD. Bars with different letters were considered significant at *p* < 0.05.

**FIGURE 5:**
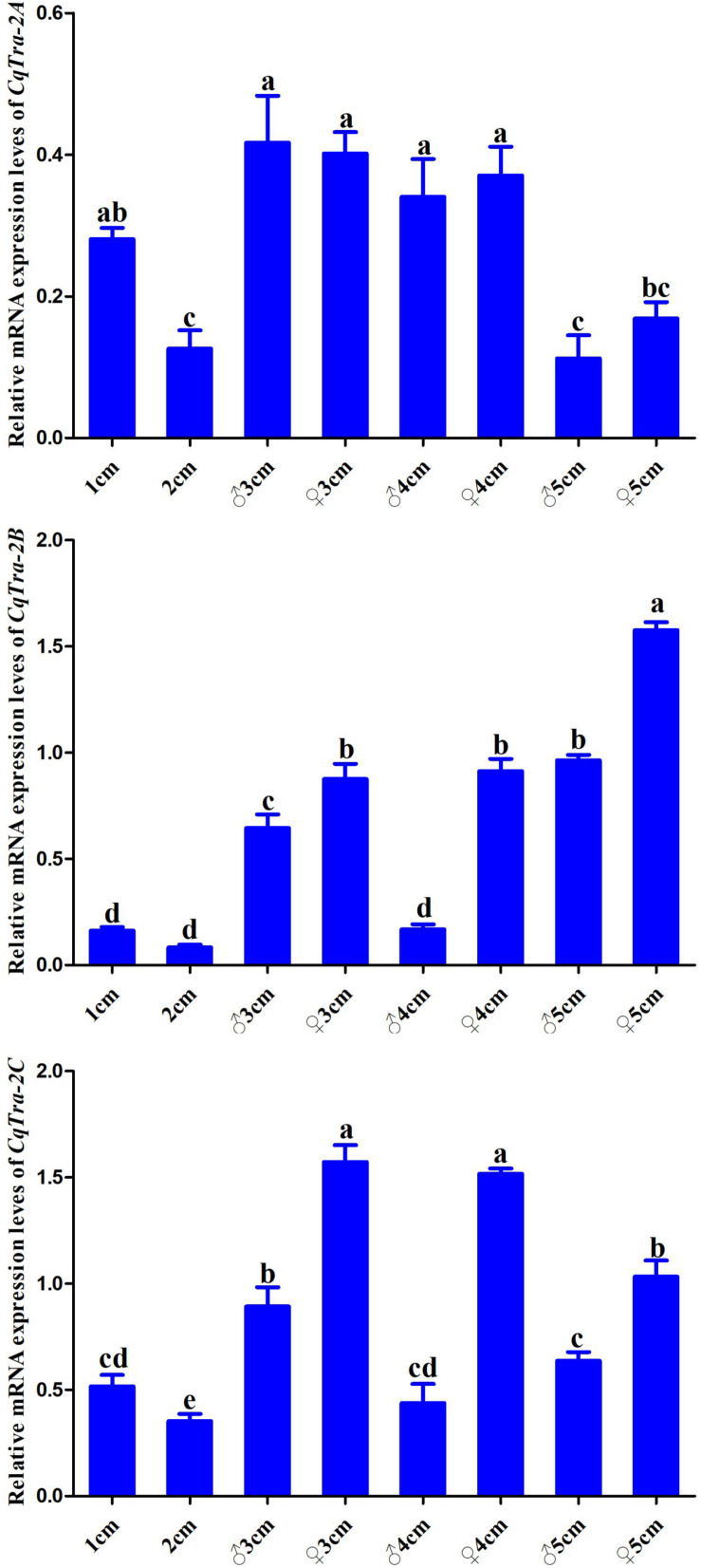
The expressions of *CqTra-2* genes analyzed by qPCR in different development stages of *C. quadricarinatus*. Numbers on the *X*-axis represent the body length of juveniles. ♀: female; ♂: male. Data are shown as means ± SD. Bars with different letters were considered significant at *p* < 0.05.

To investigate the temporal expression pattern of the *CqTra2* gene during embryogenesis and juvenile development, we measured the relative expression level at various stages using RT-PCR. All *CqTra2* transcripts were detected at a low level in fertilized eggs, at a high level in the cleavage and blastula stage, and at a peak in the prehatching stage. The mRNA expression level of *CqTra-2B* was obviously higher than *CqTra-2A* and *CqTra-2C* at each stage of embryonic development (Figure 4). Determining the initial stages of sex differentiation is crucial to study sexual regulation mechanisms. The expression levels of *CqTra-2* at different developmental stages of juveniles were analyzed by qRT-PCR. The results showed that the *CqTra-2* expression level exhibited a peak when the body length of juveniles reached 3 cm (Figure 5). Interestingly, *CqTra-2B/C* mRNA abundances were significantly different between female and male individuals, suggesting that this period may be the key point for the sex differentiation of *C. quadricarinatus.*

### The Expression Profile of *CqTra2* and *Cqdsx* After RNAi

**FIGURE 6:**
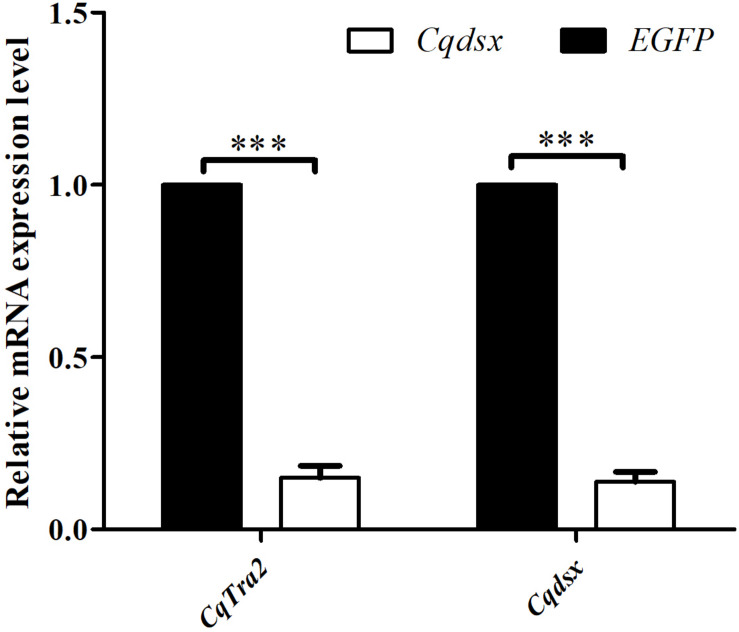
Effects of *CqTra2*-dsRNA injection on gene expression levels in cephalothoraxes of *C. quadricarinatus. EGFP*, treated with dsEGFP and used as RNAi control; *CqTra2*, treated with dsCqTra. Three individuals were pooled as one sample. Three replicates were used for analysis. Results are expressed as mean ± SEM, and significance of comparison is defined as *P* < 0.001 (***) by Student’s *t*-tests.

Considering that *CqTra-2* genes exhibited a dimorphic expression pattern in gonad tissues, we utilized RNAi to examine the function of *CqTra-2* in female sex differentiation. DNA fragments of *CqTra2* and *EGFP* containing the T7 promoter were directly synthesized and cloned into the vector for dsRNA transcription *in vitro*. An RNAi assay was performed through injection of *CqTra2*- and *EGFP-*dsRNA into the cephalothoraxes of the juveniles. The results showed that the transcription levels of *CqTra2* with the specific primers CqTra2-RT-F and CqTra2-RT-R by qRT-PCR was decreased by approximately 85%, indicating that dsRNA-mediated gene silencing was successful. In crustaceans, *doublesex* and its homologous genes were important regulators of sexual differentiation. Here, we also observed a significant decrease in *Cqdsx* after knockdown of *CqTra-2* (Figure 6). Thus, it was determined that *CqTra2* might be the upstream regulator of *Cqdsx*.

## Discussion

Sexual dimorphism is a common phenomenon in decapod aquaculture species (Sagi and Aflalo, 2005; Shi et al., 2019). Understanding the molecular mechanisms of sexual regulation in these species is attractive because a monosex culture can offer great benefits (Sagi et al., 1997; Ventura et al., 2012; Levy et al., 2016). Recently, with the development of sequencing technology, the transcriptome and genome data of some crustacean species are also being explored by researchers. Although many sex-related genes have been reported, understanding of the genetic mechanisms involved in sexual regulation in crustaceans is still limited.

In this study, the full-length cDNA sequences of three *CqTra-2* isoforms were obtained from the transcriptome library of gonadal tissues of *C. quadricarinatus*, which is a homolog of the sex differentiation protein Tra and is involved in the sex determination pathway in *D. melanogaster* (Inoue et al., 1990; Penalva and Sanchez, 2003; Sarno et al., 2010). Evidence has shown that alternative splicing plays a role in the tissue development and physiology of animals (Chen and Manley, 2009). Analysis of the partial gene organization structure of *C. quadricarinatus* showed that *CqTra-2* isoforms originated from the same genomic locus and were generated by alternative splicing of the same pre-mRNA. The *Tra2* gene has different splice variants in many arthropod species, such as *D. melanogaster* (Amrein et al., 1990), *Bombyx mori* (Niu et al., 2005), *F. chinensis* (Li et al., 2012), and *E. sinensis* (Luo et al., 2017). As shown in Figure 2A, the deduced amino acid sequences of CqTra-2A, CqTra-2B, and CqTra-2C contained a conserved RNA recognition motif (RRM), implying that the RRM domain in the CqTra-2 homolog might provide a structural basis for its potential function as a splicing factor (Luo et al., 2017). Our phylogenetic tree revealed that CqTra2 clustered with *F. chinensis*, *M. rosenbergii*, and *M. nipponense* CqTra2 and separated from two other crustaceans, *E. sinensis* and *P. monodon*. This means that the CqTra2 protein was much closer to the Tra2 protein in *F. chinensis* and diverged from that in another decapod, *P. monodon*, during evolution.

To explore the biological function of *CqTra-2* in the redclaw crayfish, the gene expression profiles of the *CqTra-2* splice variants were analyzed. First, the tissue distribution revealed that *CqTra-2* displayed a sexually dimorphic expression pattern across gonadal tissues. Unlike other species, the *CqTra-2* mRNA abundance in non-gonadal tissues of *C. quadricarinatus* was extremely low, suggesting that the primary function of *CqTra-2* was potentially to regulate gonadal development. It should also be noted that the three *CqTra-2* transcripts had a higher expression level in the ovary than in the testis, which was consistent with the expression profile of *Tra-2* in *F. chinensis* (Li et al., 2012). Moreover, *CqTra-2* gene expression was detected from fertilized eggs to the prehatching period, showing a gradual increase in early embryonic development. The zygote genome starts from the tenth cell division in zebrafish (Francisco, 2003), and we inferred that *CqTra-2* might be a maternal gene based on its mRNA expression characteristics.

To date, the sex determination mechanisms in crustacean species are unknown. RNAi is commonly used as a tool to investigate the function of target genes in many species (Hasuwa et al., 2002). Here, we employed RNAi-induced gene silencing to further explore the role of the *CqTra-2* gene in sexual regulation of *C. quadricarinatus.* After injection with *CqTra2* dsRNA, the expression level of *Cqdsx* gene was downregulated. A previous study in *F. chinensis* indicated that the *FcDsx* gene might be involved in the shrimp sexual differentiation process (Li et al., 2018). Hence, together with the expression analysis and the inhibition expression of the *CqTra2* gene, we inferred that *CqTra2* played an important role in sex differentiation. Certainly, further studies are needed to determine: (1) the role of *CqTra-2* in sex reversal or gonadal histology after RNAi treatment; (2) the interplay between *CqTra2* and *Cqdsx* in the regulation mechanism of sex differentiation.

In conclusion, we isolated and characterized three alternatively spliced isoforms of *CqTra-2* gene in *C. quadricarinatus* for the first time. Subsequently, the primary functions of *CqTra-2* were investigated using qRT-PCR and RNAi. The present data provide insights into the possible function of *CqTra-2* gene in female sex differentiation in *C. quadricarinatus*. RNAi assay revealed that *CqTra2* can regulate the expression of *Cqdsx*, indicating that *CqTra2* might be the upstream regulator of *Cqdsx*. Our results will help us to better study the sex determination mechanism in crustaceans. How the gene *CqTra-2* regulates *Cqdsx* in *C. quadricarinatus* needs further study.

## Data Availability Statement

The datasets generated for this study can be found in the MN393520, MN393521, and MN393522.

## Ethics Statement

The animal study was reviewed and approved by the Ethics Committee of Laboratory Animal Center of Zhejiang Institute of freshwater fisheries.

## Author Contributions

JZ and ZG designed the research. LC, JZ, YJ, and SL performed the experiments. JZ and LC analyzed the data. MC, LC, and SC contributed to reagents and animal materials. LC, JZ, and ZG wrote the manuscript. All authors contributed to the article and approved the submitted version.

## Conflict of Interest

The authors declare that the research was conducted in the absence of any commercial or financial relationships that could be construed as a potential conflict of interest.

## Footnotes

1 http://www.ncbi.nlm.nih.gov/gorf/orfig.cgi

2 http://www.expasy.org/

3 http://blast.ncbi.nlm.nih.gov/Blast.cgi
